# Growth of high quality and uniformity AlGaN/GaN heterostructures on Si substrates using a single AlGaN layer with low Al composition

**DOI:** 10.1038/srep23020

**Published:** 2016-03-10

**Authors:** Jianpeng Cheng, Xuelin Yang, Ling Sang, Lei Guo, Jie Zhang, Jiaming Wang, Chenguang He, Lisheng Zhang, Maojun Wang, Fujun Xu, Ning Tang, Zhixin Qin, Xinqiang Wang, Bo Shen

**Affiliations:** 1 State Key Laboratory of Artificial Microstructure and Mesoscopic Physics, School of Physics, Peking University, Beijing 100871, China; 2 Collaborative Innovation Center of Quantum Matter, Beijing 100871, China; 3 Institute of Microelectronics, Peking University, Beijing 100871, China

## Abstract

By employing a single AlGaN layer with low Al composition, high quality and uniformity AlGaN/GaN heterostructures have been successfully grown on Si substrates by metal-organic chemical vapor deposition (MOCVD). The heterostructures exhibit a high electron mobility of 2150 cm^2^/Vs with an electron density of 9.3 × 10^12^ cm^−2^. The sheet resistance is 313 ± 4 Ω/◻ with ±1.3% variation. The high uniformity is attributed to the reduced wafer bow resulting from the balance of the compressive stress induced and consumed during the growth, and the thermal tensile stress induced during the cooling down process. By a combination of theoretical calculations and *in situ* wafer curvature measurements, we find that the compressive stress consumed by the dislocation relaxation (~1.2 GPa) is comparable to the value of the thermal tensile stress (~1.4 GPa) and we should pay more attention to it during growth of GaN on Si substrates. Our results demonstrate a promising approach to simplifying the growth processes of GaN-on-Si to reduce the wafer bow and lower the cost while maintaining high material quality.

Recently, AlGaN/GaN heterostructures grown on Si substrates have attracted much attention for high power, high frequency, and high temperature applications^1^,^2^,^3^,^4^. These can offer several advantages such as large wafer size, high thermal conductivity, low cost, and great potential of the compatibility with existing processing technologies developed for Si integrated circuits^5^,^6^,^7^,^8^. Despite the promising applications, GaN-on-Si technology is facing reproducibility and reliability issues, which are likely to be related to growth processes and crystalline quality (defects and residual stress)^1^,^9^. Due to the large lattice mismatch and thermal mismatch between GaN and Si substrates, it is challenging to grow high-quality and stress-free GaN-based epilayers. Several complicated stress-control approaches such as patterned Si substrate technology^10^, LT-AlN^11^, AlN/GaN superlattice^12^,^13^, and compositionally graded AlGaN layer^14^,^15^ have been proposed to achieve crack-free GaN based heterostructures. However, the crystalline quality (defects and residual stress), as well as uniformity issues still remain, especially for growth onto large diameter substrates.

For the method with compositionally graded buffers, three step-graded AlGaN (with Al composition of about 75%, 50% and 25%) or multiple step-graded AlGaN buffers with thickness up to 1 μm are generally used^15^,^16^,^17^. The main purpose of this method is to slow down the relaxation rate of compressive stress by decreasing the lattice mismatch between the two neighbouring layers. There is thus a larger compressive stress accumulated in the GaN layer during the growth at high temperature. One issue in this case with thick buffers is that the wafer is convexly bowed. As a result, it will significantly affect the wafer uniformity during the subsequent growth. Another issue is that the growth rate of AlGaN ternary alloy is generally lower than that of GaN layer and decreases with increasing Al composition. So the growth process takes a long time in this complicated technology, and the manufacturing cost is high. It is therefore highly desirable to reduce total epilayer thickness and growth time while maintaining high material quality. For the general method with three step-graded AlGaN buffers^16^, the lattice mismatch induced compressive stress between the last AlGaN layer (with Al composition of 25%) and GaN layer is calculated to be about 2.8 GP^18^. We suppose this compressive stress value is enough to compensate the thermal tensile stress during the cooling down process which is typically reported to be about 1.4 GPa^15^. So, the questions are: whether the first two step-graded AlGaN (with Al composition of 75% and 50%) buffers are necessary for crack free GaN on Si substrate? Is it possible to get high quality GaN based materials on Si substrate using only a single AlGaN buffer layer?

In order to answer the above questions, we only keep the last AlGaN buffer layer with low Al composition in this work. By employing this low Al composition AlGaN layer as the stress-control layer, crack-free GaN buffer layers with a small wafer bow of 18 μm were obtained on 100 mm Si substrates. Nearly strain-free GaN buffer layers are obtained, thanks to the balance of the compressive stress induced by the lattice mismatch, compressive stress consumed by the dislocation relaxation, and tensile stress induced by the thermal expansion mismatch. The compressive stress consumed by the dislocation relaxation is determined to be about 1.2 GPa which is comparable to the tensile stress and should be paid more attention to during growth of GaN on Si substrate. Upon these GaN buffers, high crystalline quality and high uniformity AlGaN/GaN heterostructures with an electron mobility of 2150 cm^2^/Vs and a low sheet resistance of 313 Ω/◻ were obtained.

Figure 1(a) shows the trace of *in situ* optical reflectivity with monitoring wavelength of 950 nm during the sample growth. AlN, AlGaN, and GaN growths exhibit different oscillation periods in the figure due to their reflectivity differences at the growth temperature. No damping of the oscillation amplitude with increase of the nitride layer thickness indicates a two-dimensional growth mode and good surface morphology. The corresponding atomic force microscopic (AFM) image presents atomic-step terraces, as shown in Fig. 1(b). The root mean square roughness is 0.17 nm in a scanned area of 5 × 5 μm^2^, which indicates that the surface of the GaN layer is very smooth.

The HRXRD 2θ-ω scans for GaN (002) reflection across the sample is shown in Fig. 2. It is clearly seen that there is only one low Al composition AlGaN layer between the AlN buffer and GaN layer. The GaN layer is highly resistive due to carbon auto-doping under the low pressure growth condition, which is essential to be used as the buffer layer in the AlGaN/GaN structure^19^. The full widths of half maximum of the XRD rocking curve symmetric (002) and asymmetric (102) ω scans in the GaN layer (inset of Fig. 2) are 439 arcsec and 621 arcsec, respectively, which is among the best values for 2 μm-thick high resistance GaN layers^16^,^20^. These indicate that a 2 μm-thick high quality GaN layer can be obtained using this single AlGaN buffer.

Figure 3(a) shows the *in situ* wafer curvature data during the whole process of ramping up, growth and cooling down. The concave (convex) shaped surface of the wafer is defined as the positive (negative) curvature value. The curvature curve is divided into four processes, labelled as S1, S2, S3, and S4, respectively. In order to see the process more vividly, graphic models and cross-sectional diagrams after each growth process are also shown in Fig. 3(b). The concave curvature during process S1 is caused by the temperature gradient along the growth direction. During the process S2, the increased concave curvature with the AlN thickness indicates the tensile stress presented in the AlN buffer layer according to the Stoney’s equation^21^. During the first part of process S3, i. e. when the AlGaN intermediate layer is grown on the AlN buffer layer, the wafer curvature begins to decrease. This indicates that the compressive stress is generated in the AlGaN layer due to its larger in-plane lattice constant compared to the AlN layer. In a similar way, the GaN epilayer is also under a compressive strain condition during the second part of process S3. We observe that the curvature decreases slowly as the GaN layer is growing. This phenomenon can be explained by the fact that the compressive stress is consumed by the dislocation relaxation^22^ which will be discussed later. During the cooling down process S4, the compressive stress in the GaN layer is compensated by the thermal tensile stress, and therefore the convex wafer curvature changes towards the flat condition. The final wafer bow at room temperature is measured to be around 18 μm which meets the requirements of the CMOS standards.

In order to analyse the residual stress in the GaN layer, Raman scattering experiments were performed using the 514.5 nm line of an Argon ion laser as the excitation light source. Generally, the E_2_ (high) phonon peak in GaN is used to quantify the stress due to its sensitivity to strain/stress, as reported by F. Demangeot *et al.*^23^. A typical strain-free GaN shows E_2_ (high) phonon peak at 567.5 cm^−1 ^24. A red shift of this value is related to tensile stress, while a blue shift is related to compressive stress. To analyse the residual stress distribution across the whole wafer, Raman measurements are carried out from one edge to the other edge in the wafer. Figure 4(a) shows the Raman spectra recorded from different positions in the wafer. The position of the strain-free value (567.5 cm^−1^) is marked by the vertical short dark line near the horizontal axis. The residual stress in the GaN layer can be calculated from:


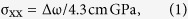


where σ_xx_ is the residual stress in the GaN layer of the measured sample, and ∆ω is the shift of E_2_ (high) phonon peak position with respect to the strain-free GaN^24^. Figure 4(b) shows the stress distribution across the sample. We can see that an average stress value in the GaN layer is in the range of −0.07 to 0.09 GPa, which indicates that the GaN layer at room temperature is almost stress-free. As shown in Fig. 3(a), the slope of the *in situ* curvature evolves into almost zero at the end of the AlGaN growth, which indicates that the AlGaN absolutely relaxes and thus almost becomes strain-free at the growth temperature. Thus, the lattice mismatch strain [(**a**_GaN_ − **a**_AlGaN_)/**a**_GaN_] between the low Al composition AlGaN layer (with Al composition of about 23%) and GaN layer can be calculated to be 0.56% in the sample. This translates to a compressive stress of 2.6 GPa in the GaN layer using the biaxial modulus of 470 GPa. Since the thermal tensile stress in GaN on Si is around 1.4 GPa, the stress difference (1.2 GPa) between the lattice mismatch stress and thermal tensile stress is mainly attributed to the dislocation relaxation. We can find that the compressive stress consumed by dislocation relaxation is comparable to the value of the thermal tensile stress. These results indicate that we must pay more attention to the compressive stress consumed by dislocation relaxation in order to finely balance the stress of GaN on Si substrate.

AlGaN/GaN heterostructures with ~1 nm-thick AlN interlayer were grown on the high quality GaN layer. Room-temperature Hall measurements was conducted by using Van der Pauw configuration on mesas with 3 × 3 mm^2^ from one wafer edge to the other one along the direction shown in Fig. 5. The two-dimensional electron gas mobility of the sample is in the range of 2030 to 2150 cm^2^/(Vs). The sheet resistance across the wafer is as low as 313 ± 4 Ω/◻, and thus the uniformity value is 1.3%. The high electron mobility and low sheet resistance indicate the high quality of the AlGaN/GaN heterostructures. AlGaN/GaN high electron mobility transistors (HEMTs) fabricated with a gate-to-source distance, gate-to-drain distance and gate length of L_GS_/L_GD_/_LG_ = 1.5 μm/3 μm/1.5 μm deliver excellent DC characteristics with a maximum drain current density (I_Dmax_) of 688 mA/mm (Fig. 6), further suggesting the high quality of the material. The above results strongly suggest that this stress control technology with only a single low Al composition AlGaN layer is efficient to improve the quality and uniformity of the AlGaN/GaN heterostructures and is important for future applications in high-power GaN based electronics devices.

In summary, using a single AlGaN layer with low Al composition, crack-free GaN epilayers with a smooth surface morphology were successfully grown on 100 mm Si substrates by MOCVD. By balancing the compressive stress induced and consumed during the growth, and the thermal tensile stress induced during the cooling down process, the GaN layers are nearly strain-free with a lower wafer bow. It is demonstrated that the stress consumed by the dislocation relaxation is comparable to the tensile stress and we should pay more attention to it during growth of GaN on Si substrate. Upon the GaN epilayers, high uniformity and high quality AlGaN/GaN heterostructures with a low sheet resistance of 313 ± 4 Ω/◻ (±1.3% variation) have been obtained. Our results open up prospects of using this technology to simplify the growth process, reduce the wafer bow and lower the epi-cost, which are important for future GaN-on-Si technology.

## Methods

### Sample preparation

The GaN layers and AlGaN/GaN heterostructures used in this work were grown on 650 μm thick 100 mm *p*-type Si (111) substrates by metal-organic chemical vapor phase deposition (MOCVD) with close coupled showerhead 3 × 2 inch reactor (Aixtron CCS). Trimethylgallium, trimethylaluminium, and ammonia were used as precursors for gallium, aluminium, and nitrogen, respectively. Hydrogen was used as the carrier gas. The Si substrates were first heated to ~1000 °C in H_2_ atmosphere and annealed for about 10 min to remove the native oxides from the surface. Next, an AlN buffer with a thickness of 270 nm was deposited at about 1100 °C followed by a single 330 nm thick AlGaN intermediate layer and a 2 μm-thick GaN layer. The AlGaN layer was grown at 1060 °C. The Al composition of the AlGaN interlayer is measured to be about 23% by high resolution X-ray diffraction (HR-XRD). All the layers were grown at a pressure of 100 mbar.

### Measurements

*In situ* wafer curvature measurements (Laytec EpiCurve^®^TT) were carried out across the sample to monitor the stress evolution during the growth and cooling down processes. Hall effect measurements in Van der Pauw configuration were taken in a Bio Rad Accent HL5500 Hall measurement system. Ohmic contacts were prepared by depositing Ti/Al/Ni/Au (20/150/35/100 nm) metal stacks followed by a rapid thermal annealing at 850 °C for 35 s under nitrogen atmosphere.

## Additional Information

**How to cite this article**: Cheng, J. *et al.* Growth of high quality and uniformity AlGaN/GaN heterostructures on Si substrates using a single AlGaN layer with low Al composition. *Sci. Rep.* **6**, 23020; doi: 10.1038/srep23020 (2016).

## Figures and Tables

**Figure 1:**
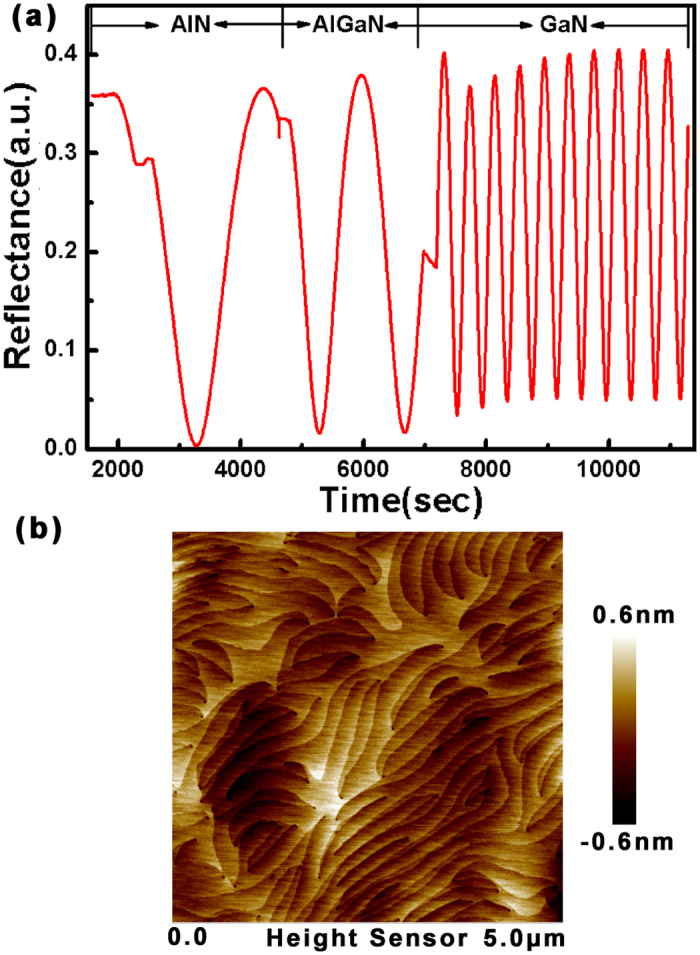
(**a**) The trace of *in situ* optical reflectivity measurement in the whole growth process. (**b**) AFM image (5 × 5 μm^2^) of the GaN layer.

**Figure 2:**
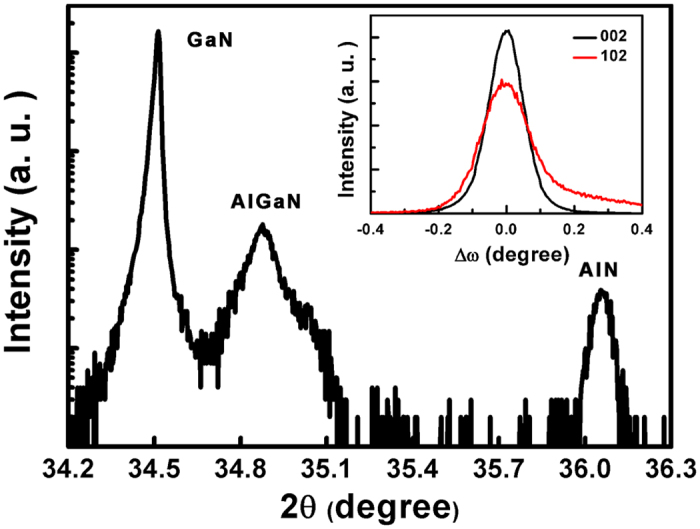
XRD 2θ-ω scans for the GaN (0002) reflection. The inset shows symmetric (002) and asymmetric (102) ω scans of rocking curve in the GaN layer.

**Figure 3:**
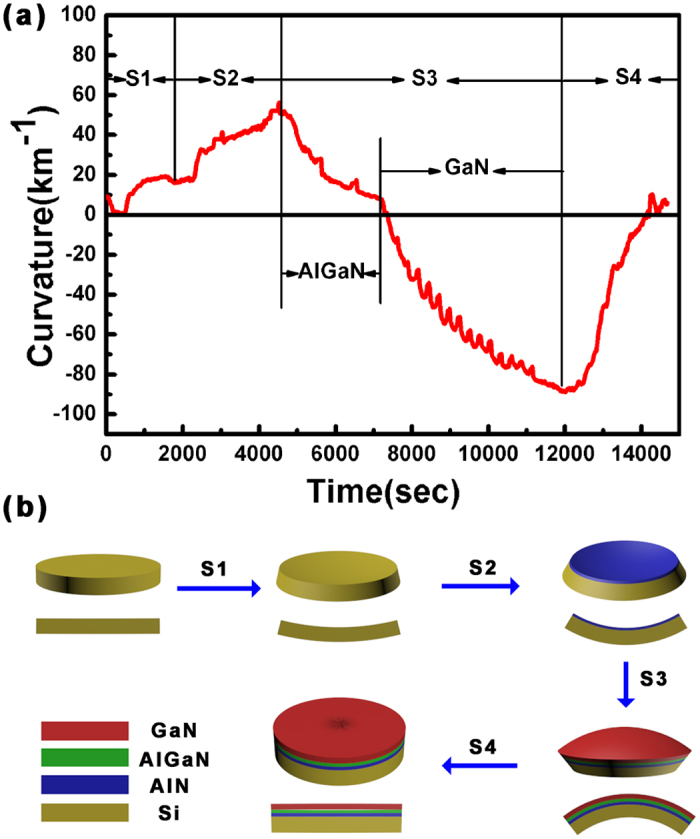
(**a**) *In situ* wafer curvature monitoring results during the growth by Laytec EpiCurve^®^TT. (**b**) Graphic models and cross-sectional diagrams of wafer bow after the processes S1, S2, S3, and S4.

**Figure 4:**
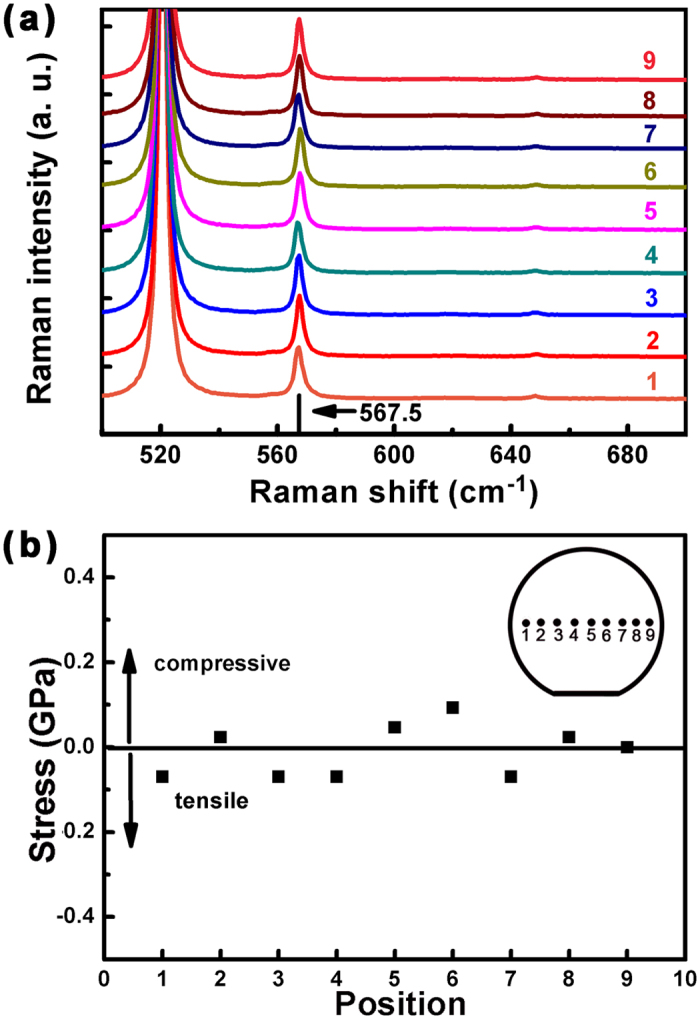
(**a**) Raman spectra recorded from different positions. (**b**) Stress distribution calculated from the Raman measurements across the sample. The measured positions are labelled by Arabic numerals (from 1 to 9) across the sample in the inset.

**Figure 5:**
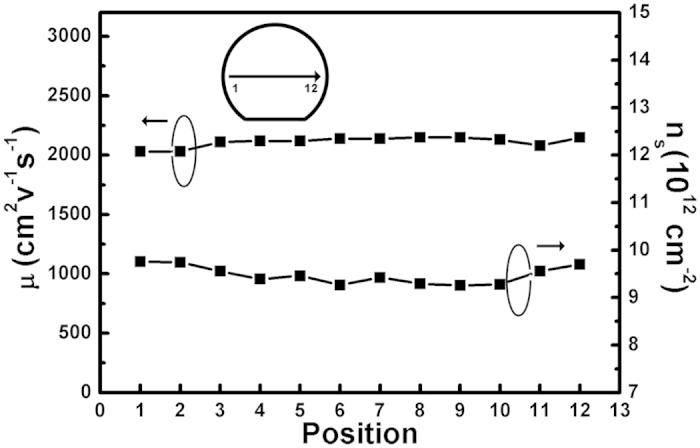
The two-dimensional electron gas density and mobility distribution across the sample. The inset shows the measured positions on the wafer.

**Figure 6:**
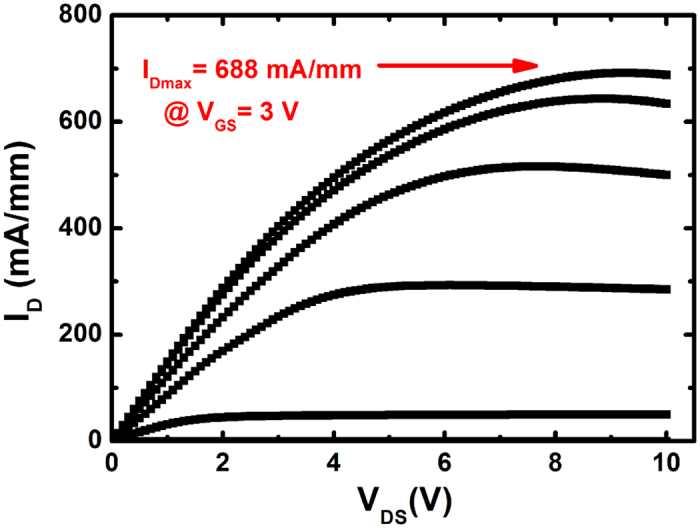
*I*_DS_-V_DS_ characteristics of AlGaN/GaN HEMTs fabricated on silicon substrates.
